# Genome-Wide Association Study for Fatty Acid Composition in American Angus Cattle

**DOI:** 10.3390/ani11082424

**Published:** 2021-08-18

**Authors:** Muhammad Dawood, Luke Matthew Kramer, Muhammad Imran Shabbir, James Mark Reecy

**Affiliations:** 1 Department of Biological Sciences, International Islamic University, Islamabad 44000, Pakistan; muhammad.dawood@uvas.edu.pk (M.D.); imran.shabbir@iiu.edu.pk (M.I.S.); 2 Department of Animal Breeding & Genetics, University of Veterinary and Animal Sciences, Lahore 54000, Pakistan; 3 Department of Animal Science, Iowa State University, 2255 Kildee Hall, Ames, IA 50011, USA; lmkramer@iastate.edu

**Keywords:** fatty acids, beef, GWAS, high density genotyping, Black Angus

## Abstract

**Simple Summary:**

Almost everybody depends on livestock for various reasons directly or indirectly. Consequently, improving livestock production means improving human life. Meat plays important role in human life, as it is good source of protein and energy. Meat composition depends on breed’s genetics and environmental factors. Fatty acids (FA) play important role in human diet and health. FA add flavor and taste to meat. Fatty acid composition of meat is a complex polygenic trait that is controlled by genetics and environmental factors. Therefore, the objective of the present study was to identify genomic regions associated with FA composition in American Angus. Thirty-six different genomic regions were identified associated with variation in at least one FA. The genomic regions associated with more than one FA and high genetic variance, harbor good candidate genes (e.g., FABP2, FASN, FADS2, FADS3 and SCD). The identified makers could be used to select for altered FA profile and help to increase the understanding of the genetic basis of FA composition. Furthermore, findings from the present study could help to devise effective breeding plans and selection strategies for the improvement of beef FA profile.

**Abstract:**

Livestock is an important commodity playing a major role in the global economy. Red meat plays an important role in human life, as it is a good source of animal protein and energy. The fatty acid content of beef has been shown to impact the eating experience and nutritional value of beef. Therefore, this study aimed to identify genomic regions which can account for genetic variation in meat fatty acid content. Genotypes imputed to the Illumina BovineHD 770K BeadChip were used in this study. Thirty-six 1-Mb genomic regions with a posterior probability of inclusion (PPI) greater than 0.90 were identified to be associated with variation in the content of at least one fatty acid. The genomic regions (1Mb) which were associated with more than one fatty acid trait with high genetic variance and harbored good candidate genes were on Chromosome (Chr) 6 (fatty acid binding protein 2), Chr 19 (thyroid hormone receptor alpha, fatty acid synthase), Chr 26 (stearoyl-CoA desaturase), and Chr 29 (thyroid hormone responsive, fatty acid desaturase 2, and fatty acid desaturase 3). Further studies are required to identify the causal variants within the identified genomic regions. Findings from the present study will help to increase understanding of the variation in fatty acid content of beef and help to enhance selection for beef with improved fatty acid composition.

## 1. Introduction

Beef has a high nutritional value, and it is a rich source of minerals, vitamins, and protein. The consumer is becoming more concerned about their health and more conscious about the quality of the meat that they consume. Consumers have been told that beef consumption is associated with some serious health issues, such as heart diseases and obesity [1]. However, recent findings have shown that the long-standing belief that beef is associated with cardiovascular disease is incorrect [2,3,4,5,6,7]. Furthermore, fat is a very important constituent of the human daily diet; it provides energy and also contains essential fatty acids, and adds flavor to food [8]. The fatty acids present in animal tissues can be separated into phospholipid and triacylglyceride fractions [9]. Fatty acid composition and fat content of the beef are associated with the taste, flavor, and sensory properties of the meat [10]. It has been reported that fatty acid composition varies across different breeds and feeding regimes [10,11].

The mechanism that control fatty acid composition of meat is a complex process that is regulated by genetics and environmental factors. There have been several studies published that evaluated the extent to which genetics controlled variation in fatty acid composition in Santa Gertrudis, Brahman, Hereford, Nellore, and Black Angus cattle breeds [12,13,14]. Identification of genomic markers and regions associated with beef fatty acids could be used to select for an improved fatty acid profile and to alter the saturated to mono- and polyunsaturated fatty acid ratios. The objective of the present study was to identify genomic regions associated with fatty acid composition in American Angus cattle.

## 2. Materials and Methods

### 2.1. Animal Selection

The purebred American Angus cattle used in the current study were reared according to standard animal care procedures, approved by the Iowa State University Animal Care and Use Committee. All the research animals were raised on Iowa State University research demonstration farms.

### 2.2. Sampling and DNA Isolation

A total of 2177 American Black Angus calves sired by 134 sires were used in this study. Blood samples were collected from the jugular vein. DNA samples were collected as previously described by Garmyn et al. [15]. DNA was stored at -20 degrees Celsius until further processing.

### 2.3. Genotype Data

Animals were originally genotyped with either the BovineSNP50 BeadChip (Illumina, San Diego, CA, USA) or the BovineHD BeadChip (Illumina, San Diego, CA, USA) by Neogen GeneSeek Operations (Lincoln, NE, USA). Animals genotyped with the BovineSNP50 BeadChip were imputed to the BovineHD BeadChip SNP density using FImpute [16] and SNPipeline package (Hailin Su, https://github.com/cbkmephisto/SNPipeline (accessed on 27 February 2017)) by using 820 Angus individuals originally genotyped on the BovineHD BeadChip. These 820 individuals included animals from the ISU herd and external animals. A filter of 0.05 minor allele frequency was applied and all markers with missing information were excluded. After filtering, a total of 199,431 markers were excluded from analysis, leaving a total of 574,662 markers for data analyses. Genome coordinates are relative to the Bovine UMD 3.1 genome assembly.

### 2.4. Fatty Acid Profile

For fatty acid profile analysis, animals were slaughtered at commercial slaughtering facilities. All the slaughtering procedures were carried out by trained personnel. Carcass data collection, tissue sampling, and fatty acid profile analysis were carried out. Fatty acid composition was analyzed as previously reported [17].

For each fatty acid, phenotypic observations were recorded on a fat percentage basis to estimate the marker effect. In this study, 56 different fatty acid traits were included.

### 2.5. Statistical Analysis

Imputed genotype data were utilized to estimate the SNP effect associated with fatty acid composition. Statistical analysis was performed using the BayesB method for genomic prediction [18]. Data were analyzed via the following model:

y = Xb + Zu + e

where y is the observable value for fatty acid, and X and Z are fixed and random effects, respectively. In this model, b is the fixed effect (age, sex, and population mean), u is the random effect marker, and e is the residual effect [18,19]. Fixed effect and covariates included: contemporary group, sex and hot carcass weight, longissimus muscle area at 12th rib, subcutaneous fat thickness at 12th rib and chemically extracted fat. All the analyses were performed using GenSel software [20].

A chain of 50,000 iterations with the first 5000 as burn-in was used, and the parameter pi (π) was set at 0.99906 (99.9%; approximately 540 SNP markers with a non-zero effect), while genetic and residual variances for each trait were estimated using BayesC (initial variances set as half the total phenotypic variance) before being used in BayesB [21,22]. The posterior probability of inclusion and correlation between QTL and trait were calculated, as described by [20].

## 3. Results and Discussion

### 3.1. Fatty Acid Data Statistics

Summary statistics for the studied fatty acids traits are given in Table 1.

### 3.2. Posterior Residual and Genetic Variance, Heritability Estimation

The SNP-based heritability estimates for fatty acid traits in this study ranged from 0.005 to 0.610 (Table 2). The lower heritability estimates indicated that SNP markers would be poor predictors of fatty acid composition, whereas the higher heritability estimates demonstrate that SNP markers could provide reliable predictions for the content of some fatty acids. The highest heritability estimate was 0.610 for C13:0 and 0.478 for C18:1 trans-12. There were ten fatty acid traits that appeared to have moderate heritability values. These ten fatty acids and their respective h^2^ values were C16:0 (0.20), LCFA (0.231), MCFA (0.232), C14:1 (0.232), n3/n6 (0.237), C14:0 (0.262), C16:1/C16:0 (0.266) C16:0/C14:0 (0.286), C20:0 (0.298), and UFA/SFA (0.359). Heritability estimates for all fatty acid traits are reported in Table 2.

All other traits (excluding those described above) appeared to have low heritability values. For these traits, the amount of phenotypic variance explained by the markers was low. The lowest heritability estimated was on fatty acid ratio C18:0/C16:0 (0.005). Previously reported heritability values for fatty acid traits were lower than in the present study. In American Angus, using 50k SNP chip data, the highest heritability value (0.57) was reported for saturated fatty acid C14:0 [18], while in Nellore cattle, the highest reported value was 0.24 for C17:0 and C18:3-n6 [12]. Another study in Canada on beef cattle showed higher h^2^ values of 0.57 and 0.59 for a saturated fatty acid (C17:0) and monounsaturated fatty acids (C14:1 and C18:1) [23]. A previous study showed that analysis of fatty acid content on a fat percentage basis was able to explain a greater proportion of phenotypic variance by SNP markers, as compared to using fatty acid content on a beef basis [18].

### 3.3. Genome-Wide Association Study

A total of 56 fatty acid traits including saturated, monounsaturated, polyunsaturated, and fatty acid groups were used for genome-wide association studies. The identified genomic regions (1-Mb windows) that showed high genetic variance and posterior probability of inclusion (PPI) greater than 90% for having non-zero genetic variance or above are presented in Table 3.

The highest estimated genetic variance explained by a single SNP window was 88.09% for the fatty acid C22:1, while the window with the lowest estimated genetic variance, 1.72%, was for fatty acid C18:1t12. Regarding the SNP window which explained the highest level of genetic variance (30_72), there was a potential candidate gene, phosphatidylinositol specific phospholipase C X domain containing 1 (*PLCXD1*), on the pseudo-autosomal region (PAR). This gene is X-linked in ruminants. *PLCXD* gene products are phosphodiesterases involved in the regulation of cytosolic calcium and have protein kinase activity [24,25].

This study identified a total of 36 different 1-Mb SNP windows that were associated with fatty acid content of skeletal muscle. Three windows (19_51, 26_21, and 29_18) appeared to be associated with most beef fatty acid traits. These three windows were previously reported in the same Black Angus population using the Bovine SNP50 BeadChip [18]. Many of the 1-Mb SNP windows were associated with more than one fatty acid trait. A genomic region on chromosome 19 (at 51 Mb) was associated with 15 fatty acid traits (LCFA, MCFA, MUFA, MUFA/SFA, SFA, UFA/SFA, C14:0, C14:1, C16:0, C16:1, C18:1c9, C16:0/C14:0, C16:1/C16:0, C16:1-C18:1/C16:0-C18:0, and C18:0/C16:0). This region was also previously reported to be associated with important fatty acid traits [18,26]. This SNP window contains a good candidate gene—fatty acid synthase (*FASN*). This gene has been reported to be involved in beef fatty acid composition [27]. It has also been reported to be associated with adipose composition, milk fatty acid composition, and milk fat content in many different breeds of cattle. These reports indicated that this gene has a pivotal role and is an important candidate gene for fatty acid composition [26,28,29,30,31].

Similar to the window at 51 Mb on chromosome 19, there were additional SNP windows that were associated with more than ten fatty acid traits, including a window at 21 Mb on Chromosome 26, which was associated with 13 FA traits, and a window at 18 Mb on Chromosome 29, which was associated with 11 FA traits. These two SNP windows were also previously reported to be associated with various FA traits [18,26]. These two regions harbor good candidate genes for fatty acid composition, including stearoyl-CoA desaturase (*SCD*) and thyroid hormone responsive (*THRSP*). Previous studies have reported that SCD is associated with meat fat composition and milk fat composition [28,29,30,31]. Thyroid hormone responsive and stearoyl-CoA desaturase is involved in fatty acid synthesis [32]. Variants in the *THRSP* gene have been shown to be associated with the synthesis of beef fatty acids, which are expected to have a direct impact on beef quality [18,33]. It has also been reported that both *SCD* and *THRSP* genes are involved in lipid metabolism in cattle [34]. The 1-Mb SNP windows that harbor good candidate genes for fatty acid synthesis and fat regulation are shown in Table 4.

In the present GWAS, some new genomic windows were identified which were not previously reported by any study. These new SNP windows are associated with different fatty acid traits and harbor good candidate genes for fatty acid composition. On chromosome 16, a 1-Mb SNP window at 4 Mb was associated with SFA, MUFA, MUFA/SFA, UFA/SFA, and C16:1, C18:1/C16:0, C18:0. This window contains good candidate genes—6-phosphofructo-2-kinase/fructose-2,6-biphosphatase 2 (*PFKFB2*) and peptidase M20 domain-containing 1 (*PM20D1*). The *PFKFB2* gene has a role in degradation and synthesis of fructose-2,6-biphosphate [26]. It has been previously reported that a QTL spanning this region is related to fat thickness at the 12th rib in American Angus [35]. Peptidase M20 domain-containing 1 (*PM20D1*) is an enzyme that synthesizes N-acyl amino acids (NAAs). NAAs are bioactive lipids composed of fatty acyl chains. *PM20D1* regulates the condensation and hydrolysis of N-acyl amino acids from free amino acids and fatty acids [36,37,38]. Fatty acid binding protein 2 (*FABP2*) may have a role in lipogenesis and adipose tissue weight variability [39]. This gene has not been identified in previous GWAS as having a significant association with fatty acid composition [40]. Bardet-Biedl syndrome 4 (*BBS4*) is involved in the secretion and expression of Follistatin-like 1 (*FSTL1*), which is associated with adipogenesis. *BBS4* also plays a role in fatty acid profile, lipolysis, and fat accumulation [41,42]. Acetyl-CoA acyltransferase 2 (*ACAA2*) codes for an enzyme from the thiolase family. This enzyme is involved in elongation and degradation of fatty acids. It has been associated with milk yield and fat yield in dairy sheep [43]. Fatty acid desaturase 2 (*FADS2*) and fatty acid desaturase 3 (*FADS3*) belong to the fatty acid desaturase family. This family of genes creates a cis double bond in FA chains at specific sites and is associated with desaturation of fatty acids and blood phospholipids [44,45]. Oxysterol binding protein like 5 (*OSBPL5*) is a lipid transporter, chiefly linked with the exchange of phosphatidylserine with phosphatidylinositol 4-phosphate. It has a role in maintaining cholesterol balance [46,47].

These genes have not been previously identified by any GWAS as being related to fatty acid content and fat regulation. In the present GWAS, we did not identify any SNP windows which contain some genes (*LXR*, *LXRA*, *SREBP1*, *PPARG*, *ACSL1*, *LEP*, *ACACA*, *FABP4*, and *SL1TRK6*) previously shown to be associated with FA composition and variation in beef cattle [27,48,49,50,51,52,53]. This may indicate that genetic control of fatty acid content varies greatly from breed to breed.

### 3.4. Correlation within Genomic Regions

All of the identified regions of the genome had a genetic correlation with a fatty acid trait that ranged from 0.1 to 0.74. Both the highest genetic correlation of 0.74 and the lowest genetic correlation of 0.1 were observed for C13:0 (6_95 and 6_65). These genomic windows lack candidate genes associated with fatty acid composition or fat regulation. Some genomic regions (6_7, 10_19, 16_4, 19_42, 19_51, 24_49, 26_21, 29_18, 29_42, and 29_49) that had high correlations with fatty acid content do contain potential candidate genes (*FABP2*, *BBS4*, *PFKFB2*, *THRA*, *FASN*, *ACAA2*, *SCD*, *THRSP*, *FADS2*, *FADS3*, *MOB2*, *OSBPL5*). Three of the genomic regions (19_51, 26_21, 29_18) were previously reported by our group to have a high correlation with fatty acid content [18]. These genomic windows harbor good candidate genes (*FASN*, *SCD*, *THRSP*) that may be associated with fatty acid composition [27,28,49,54,55]. Besides these three genomic windows, there are other genomic regions (6_7, 10_19, 16_4, 19_42, 24_49, 29_42, and 29_49) that had a positive correlation with different saturated or monounsaturated fatty acid traits (C14:0, C14:1, C16:1, C17:0, MCFA, LCFA) and fatty acid groups (C16:0/C14:0 and C16:1/C16:0). These regions have not been previously reported to be associated with fatty acid content, but they contain possible candidate genes (*FABP2*, *BBS4*, *THRA, ACAA2, FADS2*, and *FADS3*) for fatty acid synthesis/composition and fat regulation. [39,40,44,45,56,57,58,59,60,61,62,63,64].

## 4. Conclusions

Genome-wide association studies can provide insight into understanding the mechanisms underlying fatty acid composition. Furthermore, genomic selection methodology can be used to select for, and to alter, fatty acid content. This study utilized imputed BovineHD BeadChip (770k) genotypes along with skeletal muscle fatty acid content phenotypic data to identify 36 1-Mb SNP windows that had a PPI > 0.90. Some of these SNP windows have been previously reported, including 19_51, 26_21, and 29_18. In addition, some new genomic regions that had not been previously reported to be associated with fatty acid content were identified: 6_7, 19_42, and 29_42. Fatty acid composition and deposition are complex polygenic traits having low to moderate heritability. The genomic regions identified in the present study and associated potential candidate genes for FA composition could help increase understanding of the genetic basis of FA composition in beef cattle (Black Angus). This study could also help to devise sensible breeding plans and selection strategies based on identified genomic regions for the improvement of beef fatty acid profile.

## Figures and Tables

**Table 1:** Fatty acids and fatty acids groups statistics summary (mean, standard deviation, and coefficient of variance) for all the studied traits.

Trait	Mean	SD	CV%
C10:0	0.04	0.07	196.81
C12:0	0.06	0.06	90.09
C13:0	0.01	0.07	1105.43
C14:0	2.71	0.58	21.21
C14:1	0.57	0.20	34.78
C15:0	0.59	0.33	55.61
C16:0	26.57	1.80	6.79
C16:1	3.49	0.71	20.38
C17:0	1.34	0.39	29.14
C17:1	1.07	0.37	34.65
C18:0	13.62	1.91	14.01
C18:1 c9	38.55	2.79	7.24
C18:1 c11	0.10	0.10	106.32
C18:1 c12	0.25	0.16	63.85
C18:1 c13	0.10	0.10	105.96
C18:1 t6/9	0.13	0.23	178.88
C18:1 t10/11	3.58	1.39	38.84
C18:1 t12	0.07	0.24	355.96
C18:1 t15	1.03	0.51	48.92
C18:2	3.94	1.31	33.33
CLA c9t11	0.13	0.13	104.68
CLA t10c12	0.05	0.09	174.15
C18:3 n3	0.17	0.16	92.47
C18:3 n6	0.02	0.03	222.57
C20:0	0.02	0.04	178.69
C20:1	0.09	0.11	116.14
C20:2	0.04	0.05	131.67
C20:3 n3	0.02	0.09	378.10
C20:3 n6	0.12	0.17	139.19
C20:4	0.77	0.38	48.84
C20:5	0.13	0.29	215.61
C22:0	0.11	0.15	135.98
C22:1	0.01	0.06	1073.49
C22:4	0.06	0.14	215.40
C22:5	0.13	0.17	127.20
C22:6	0.08	0.16	194.83
C23:0	0.07	0.18	256.19
C24:0	0.14	0.37	258.53
SFA	45.29	2.39	5.27
MUFA	49.04	2.79	5.70
PUFA	5.67	1.85	32.66
MCFA	3.98	0.80	20.02
LCFA	96.02	0.80	0.83
n3	0.54	0.55	101.90
n6	5.13	1.64	32.04
n3/n6	0.11	0.13	117.97
AI	0.69	0.09	12.91
PUFA/SFA	0.13	0.04	34.10
UFA/SFA	1.21	0.12	9.72
MUFA/SFA	1.09	0.11	10.35
C14:1/C14:0	0.21	0.05	25.99
C16:0/C14:0	10.19	2.05	20.07
C16:1/C16:0	0.13	0.02	18.66
C17:1/C17:0	0.88	1.50	170.85
C18:0/C16:0	0.52	0.09	16.80
C16:1-C18:1/C16:0-C18:0	1.18	0.12	10.28

**Table 2:** Posterior residual variance (σ^2^_e_, g × 10^−10^) estimate, genetic variance (σ^2^_g_, g × 10^−10^) estimate and the estimated heritability (h^2^) for fatty acids (Fat% basis).

Trait	σ^2^_e_, g × 10^−10^	σ^2^_g_, g × 10^−10^	h^2^
SFA	3.995	1.029	0.200
C10:0	0.005	0.000	0.072
C12:0	0.003	0.000	0.021
C13:0	0.002	0.003	0.610
C14:0	0.226	0.080	0.262
C15:0	0.071	0.002	0.023
C16:0	2.267	0.568	0.200
C17:0	0.066	0.005	0.077
C18:0	2.133	0.426	0.166
C20:0	0.001	0.000	0.298
C22:0	0.012	0.000	0.023
C23:0	0.031	0.001	0.019
C24:0	0.099	0.012	0.111
MUFA	4.932	0.879	0.151
C14:01	0.023	0.007	0.232
C16:1	0.420	0.083	0.165
C17:1	0.058	0.003	0.045
C18:1 cis-9	5.390	1.089	0.168
C18:1 cis-11	0.011	0.000	0.016
C18:1 cis-12	0.018	0.002	0.104
C18:1 cis-13	0.012	0.000	0.015
C18:1 trans-6/9	0.044	0.006	0.121
C18:1 trans-10/11	1.397	0.103	0.068
C18:1 trans-12	0.032	0.029	0.478
C18:1 trans-15	0.217	0.006	0.029
C20:1	0.004	0.000	0.014
C22:1	0.004	0.000	0.102
PUFA	2.404	0.067	0.027
C18:02	1.228	0.043	0.034
C18:3 n-3	0.014	0.000	0.019
C18:3 n-6	0.001	0.000	0.016
C20:2	0.002	0.000	0.016
C20:3 n-3	0.009	0.000	0.014
C20:3 n-6	0.025	0.002	0.062
C20:4	0.109	0.003	0.025
C20:5	0.070	0.008	0.105
C22:4	0.015	0.001	0.045
C22:5	0.017	0.000	0.018
C22:6	0.019	0.004	0.191
CLA c9t11	0.015	0.000	0.025
CLA t10c12	0.007	0.000	0.042
n-3	0.190	0.027	0.124
n-6	1.942	0.057	0.029
n-3/n-6	51.527	15.985	0.237
AI	11.039	1.014	0.084
MCFA	0.444	0.134	0.232
LCFA	0.445	0.133	0.231
MUFA/SFA	0.012	0.002	0.134
PUFA/SFA	3.856	0.887	0.187
UFA/SFA	0.007	0.004	0.359
C14:1/C14:0	0.001	0.001	0.026
C16:0/C14:0	2.290	0.916	0.286
C16:1/C16:0	0.000	0.000	0.266
C16:1,C18:1/C16:0,C18:0	0.013	0.002	0.131
C17:1/C17:0	0.058	0.003	0.045
C18:0/C16:0	0.004	0.002	0.005

**Table 3:** 1-Mb SNP windows with PPI 90% or above for fatty acids on fat percent basis.

Trait	PPI ^1^	BTA_Mb	Start SNP–End SNP	SNP	Var %	Map Position
C10:0	0.944	10_99	rs381994440–rs1116598207	258	16.78	99005624–99999474
C13:0	0.987	21_10	rs445662296–rs722133270	249	7.88	10001943–10995552
	0.989	6_65	rs723554673–rs377954800	159	41.63	65014180–65999168
	0.989	6_66	rs461561099–rs721295607	237	33.69	66005071–66997033
	0.942	6_95	rs525139052–rs714396520	250	3.24	95004561–95991513
C14:0	1	10_19	rs457389817–rs721999834	231	9.29	19000816–19989351
	1	19_51	rs475360660–rs383058850	204	54.85	51028723–51996481
	1	26_21	rs1118223446–rs475475724	186	7.39	21002029–21996318
	1	29_18	rs136831403–rs438026448	82	23.08	18005978–18986358
C14:1	1	10_19	rs457389817–rs721999834	231	14.66	19000816–19989351
	1	19_51	rs475360660–rs383058850	204	34.52	51028723–51996481
	1	26_21	rs1118223446–rs475475724	186	20.19	21002029–21996318
	1	29_18	rs136831403–rs438026448	82	18.14	18005978–18986358
C16:0	1	19_51	rs475360660–rs383058850	204	49.93	51028723–51996481
	1	29_18	rs136831403–rs438026448	82	21.78	18005978–18986358
C16:1	0.966	10_19	rs457389817–rs721999834	231	7.78	19000816–19989351
	1	19_51	rs475360660–rs383058850	204	26.23	51028723–51996481
	1	26_21	rs1118223446–rs475475724	186	13.85	21002029–21996318
	0.998	29_18	rs136831403–rs438026448	82	16.12	18005978–18986358
C17:0	0.984	19_42	rs137786121–rs466240300	251	22.28	42004863–42996553
	1	24_49	rs381050710–rs477123921	155	33.23	49001908–49998425
C17:1	0.984	24_49	rs381050710–rs477123921	155	37.85	49001908–49998425
C18:0	0.92	26_21	rs1118223446–rs475475724	186	7.98	21002029–21996318
	1	29_18	rs136831403–rs438026448	82	17.1	18005978–18986358
C18:1 c9	1	19_51	rs475360660–rs383058850	204	50.1	51028723–51996481
	1	29_18	rs136831403–rs438026448	82	12.23	18005978–18986358
C18:1 c12	1	26_21	rs1118223446–rs475475724	186	49.36	21002029–21996318
C18:1 t 6/9	0.944	27_39	rs451168763–rs381689313	260	8.32	39005538–39990529
C18:1 t10/11	0.996	20_4	rs721326040–rs460617564	255	23.47	4010324–4999564
C18:1 t12	0.922	10_96	rs479600948–rs109335292	256	2.49	96019445–96996211
	1	16_46	rs478465218–rs797599032	224	11.46	46008112–46996022
	1	16_48	rs135228863–rs474907119	317	9.63	48017181–48984950
	0.98	17_65	rs378071414–rs525333053	285	7.51	65009493–65999327
	1	21_59	rs472316688–rs451806225	389	2.81	59000097–59996736
	1	5_111	rs468287514–rs444667395	276	5.12	111007549–111997116
	0.902	5_9	rs516462777–rs1116817234	152	11.55	9015235–9989218
C20:3 n6	0.924	3_86	rs718706801–rs730733704	264	55.3	86003522–86991729
C20:5	1	29_49	rs472519303–rs526164614	93	22.54	49009465–49997333
	0.991	3_86	rs718706801–rs730733704	264	55.49	86003522–86991729
C22:1	0.993	21_10	rs445662296–rs722133270	249	14.33	10001943–10995552
	0.987	30_71	rs458478290–rs481059659	65	36.65	71227458–71976081
	0.987	30_72	rs524807927–rs135609351	29	88.09	72004959–72982639
C22:4	0.924	25_11	rs467215611–rs456314684	387	27.95	11001504–11999813
C22:6	0.96	25_11	rs467215611–rs456314684	387	6.24	11001504–11999813
	1	3_86	rs718706801–rs730733704	264	76.04	86003522–86991729
C24:0	1	16_65	rs468954509–rs1117996716	305	50.06	65001270–65996849
	0.946	3_86	rs718706801–rs730733704	264	39.59	86003522–86991729
SFA	0.949	1_115	rs436612027–rs715205098	224	6.43	115000891–115995052
	0.998	16_4	rs450830345–rs379811569	313	6.69	4004764–4992166
	1	19_51	rs475360660–rs383058850	204	31.53	51028723–51996481
	1	26_21	rs1118223446–rs475475724	186	12.23	21002029–21996318
	0.951	7_93	rs443092875–rs378089989	143	6.46	93002992–93993941
MUFA	0.926	16_4	rs450830345–rs379811569	313	5.63	4004764–4992166
	1	19_51	rs475360660–rs383058850	204	35.94	51028723–51996481
	0.913	26_21	rs1118223446–rs475475724	186	9.54	21002029–21996318
MCFA	1	10_19	rs457389817–rs721999834	231	11.22	19000816–19989351
	1	19_51	rs475360660–rs383058850	204	57.4	51028723–51996481
	1	29_18	rs136831403–rs438026448	82	23.19	18005978–18986358
LCFA	1	10_19	rs457389817–rs721999834	231	11.35	19000816–19989351
	1	19_51	rs475360660–rs383058850	204	57.3	51028723–51996481
	1	29_18	rs136831403–rs438026448	82	23.02	18005978–18986358
n3	1	29_49	rs472519303–rs526164614	93	11.73	49009465–49997333
	1	3_86	rs718706801–rs730733704	264	76.51	86003522–86991729
UFA/SFA	0.982	1_115	rs436612027–rs715205098	224	3.92	115000891–115995052
	0.991	16_4	rs450830345–rs379811569	313	3.24	4004764–4992166
	1	19_51	rs475360660–rs383058850	204	15.35	51028723–51996481
	0.962	22_9	rs446574361–rs134422456	247	1.87	9004252–9998969
	0.989	26_21	rs1118223446–rs475475724	186	5.34	21002029–21996318
	0.931	26_32	rs136160709–rs382889271	271	2.06	32010478–32984426
	0.993	7_93	rs443092875–rs378089989	143	3.86	93002992–93993941
MUFA/SFA	0.989	16_4	rs450830345–rs379811569	313	5.27	4004764–4992166
	1	19_51	rs475360660–rs383058850	204	35.99	51028723–51996481
	0.904	26_21	rs1118223446–rs475475724	186	7.44	21002029–21996318
C14:1/C14:0	1	26_21	rs1118223446–rs475475724	186	52	21002029–21996318
C16:0/C14:0	1	19_51	rs475360660–rs383058850	204	34.55	51028723–51996481
	1	26_21	rs1118223446–rs475475724	186	6.99	21002029–21996318
	1	29_18	rs136831403–rs438026448	82	15.52	18005978–18986358
	0.947	29_42	rs379690091–rs463588285	155	5.38	42001720–42991376
C16:1/C16:0	0.984	10_19	rs457389817–rs721999834	231	5.7	19000816–19989351
	0.989	19_51	rs475360660–rs383058850	204	6.14	51028723–51996481
	1	26_21	rs1118223446–rs475475724	186	8.4	21002029–21996318
	0.96	26_29	rs380753352–rs42912734	240	3.41	29000086–29997851
	0.964	29_18	rs136831403–rs438026448	82	4.26	18005978–18986358
	0.935	6_7	rs43449965–rs797424312	218	3.36	7050818–7999410
C18:0/C16:0	0.824	15_75	rs474144146–rs799862808	288	2.87	75000095–75998710
	1	19_51	rs475360660–rs383058850	204	13.38	51028723–51996481
	0.9	21_22	rs449389929–rs437752920	258	2.43	22011632–22997995
	1	29_18	rs136831403–rs438026448	82	15.26	18005978–18986358
C16:1,C18:1/ C16:0,C18:0	0.995	16_4	rs450830345–rs379811569	313	5.62	4004764–4992166
	1	19_51	rs475360660–rs383058850	204	31.51	51028723–51996481

^1^ PPI = Posterior Probability of Inclusion.

**Table 4:** 1Mb chromosome windows having candidate gene associated with fatty acid traits.

Sr.	Region (BTA_Mb)	Gene	Traits
1	6_7	FABP2	C16:1/C16:0
2	10_19	BBS4	C14:0, C14:1, C16:1, MCFA, LCFA, C16:1/C16:0
3	16_4	PFKFB2, IL10, RAB7B, PM20D1	SFA, MUFA, MUFA/SFA, UFA/SFA, C16:1,C18:1/C16:0,C18:0
4	19_42	THRA	C17:0
5	19_51	FASN	C14:0, C14:1, C16:0, C16:0/C14:0, C16:1, C16:1/C16:0, C18:0/C16:0, C18:1 c9, LCFA, MCFA, MUFA, MUFA/SFA, SFA, UFA/SFA, C16:1,C18:1/C16:0,C18:0
6	24_49	ACAA2	C17:0, C17:1
7	26_21	SCD	C14:0, C14:1, C14:1/C14:0, C16:0/C14:0, C16:1, C16:1/C16:0, C18:0, C18:1 c12, MUFA, MUFA/SFA, SFA, UFA/SFA
8	29_18	THRSP	C14:0, C14:1, C16:0, C16:0/C14:0, C16:1, C16:1/C16:0, C18:0, C18:0/C16:0, C18:1 c9, LCFA, MCFA
9	29_42	FADS2, FADS3	C16:0/C14:0
10	29_49	MOB2, INS, OSBPL5	C20:5, n3

## Data Availability

Data will be available upon request.
